# Fig Macula as a Key Multifunctional Structure Mediating the Fig–Fig Wasp Mutualism

**DOI:** 10.3390/plants14182885

**Published:** 2025-09-17

**Authors:** Simone Pádua Teixeira, Jackeline Varanda Silva, Vitor Cassius Santos, Luan Mazzeo, Rayssa Conceição Coelho Correa, Rodrigo Augusto Santinelo Pereira

**Affiliations:** 1 Departamento de Ciências Farmacêuticas, Faculdade de Ciências Farmacêuticas de Ribeirão Preto, Universidade de São Paulo, Ribeirão Preto 14040-903, SP, Brazil; spadua@fcfrp.usp.br (S.P.T.); jackelinegm7@usp.br (J.V.S.); vitorcsantos@usp.br (V.C.S.); 2 Departamento de Biologia, Faculdade de Filosofia, Ciências e Letras de Ribeirão Preto, Universidade de São Paulo, Ribeirão Preto 14040-130, SP, Brazil; luanmazzeo@usp.br (L.M.); rayhcoelho20@usp.br (R.C.C.C.)

**Keywords:** extrafloral nectary, glands, insect–plant interaction, microscopy, mutualism, odoriferous nectar, pollination

## Abstract

Plant-insect mutualisms often drive the evolution of adaptive morphological and physiological traits, enabling ecological specialization and diversification. Fig trees (*Ficus* spp., Moraceae) and their pollinating wasps (Agaonidae) are engaged in a brood-site pollination mutualism that exemplifies such adaptive specializations. This study investigates the morphological and ecological roles of maculae, characterized as distinct-pigmented regions on the fig surface, in the mutualistic interaction between *Ficus citrifolia* and fig wasps. Through morphological analyses using light and electron microscopy, we demonstrated that maculae concentrate numerous stomata and exhibit secretory activity. This activity is evidenced by the exudation of a sugary-like solution and by the presence of epidermal and subepidermal cells with features consistent with sugar- and terpene-secreting cells, such as abundant starch reserves, numerous mitochondria, plastids containing osmiophilic droplets, a Golgi complex with dilated cisternae, oil bodies, and extensive endoplasmic reticulum. Histochemical tests confirmed a terpenic-sugary secretion in the macula cells. We demonstrated that non-pollinating fig wasps avoid ovipositing through macular regions. This behavior may reflect a selective pressure to prevent structural damage to maculae caused by ovipositor insertion, thus preserving their functional integrity. Temperature measurements revealed that figs are up to 10% cooler on average than the ambient air. Therefore, our findings suggest that fig maculae are multifunctional structures, simultaneously performing the roles of extrafloral nectaries, gas exchange, and thermal regulation, which are crucial for maintaining suitable internal conditions for wasp larval development. These results provide novel insights into previously underexplored plant adaptations supporting specialized brood-site pollination mutualisms.

## 1. Introduction

It is widely accepted that insect–plant interactions are fundamental drivers of terrestrial biodiversity, shaped by reciprocal selective pressures between animals and their associated plants, often leading to adaptive diversification [1,2,3,4]. Reciprocal selective pressures are evidenced, for instance, by plant structures such as extrafloral nectaries and domatia, as well as floral traits, which are functionally related to maintaining mutualistic interactions with insects that protect plants or facilitate their reproduction [5,6,7,8]. However, less evident and comparatively understudied are other physiological and morpho-functional plant adaptations essential to enabling mutualistic plant–insect interactions, particularly in symbiotic mutualisms. A notable example is the highly specialized symbiotic mutualism between epiphytic plants of the genus *Squamellaria* Becc. (Rubiaceae) and ants [*Philidris nagasau* (Mann), Dolichoderinae]. These plants produce a highly modified domatia derived from hypocotyls. These domatia have internal hollow galleries with two structurally distinct regions: warted walls for nutrient absorption from ant waste and smooth walls serving as brood-rearing sites [9]. Another illustrative example involves physiological adaptations in thermogenic plants, such as *Rafflesia* spp. (Rafflesiaceae) and *Helicodiceros muscivorus* (L. f.) Engl. (Araceae), which are pollinated by necrophagous flies. Their floral structures mimic decaying carcasses, and floral heating reinforces this mimicry by raising the temperature to levels comparable to rotting flesh, and enhancing the volatilization of fetid odors that attract the insects [10,11]. In thermogenic plants, heat is generated through intense cellular respiration, resulting in mass-specific rates of O_2_ consumption of up to 0.9 mol s^−1^ g^−1^, comparable to the oxygen consumption rates observed in insect flight muscle [12,13].

Fig trees (*Ficus* L., Moraceae) constitute an ideal model group for investigating morphological adaptations related to specialized mutualistic interactions. These plants have a unique, urn-shaped inflorescence called a syconium (or fig), which encloses numerous flowers internally. Because the flowers are enclosed within the fig, they lack direct contact with the external environment, with the only connection provided by a narrow apical pore called the ostiole, sealed by tightly overlapping bracts. Pollination occurs exclusively through an obligate mutualism with fig wasps (Hymenoptera, Agaonidae), whose females are morphologically adapted to enter the fig, carrying pollen into its internal cavity to pollinate the flowers [14]. This highly specialized interaction is classified as brood-site pollination, as the primary resource offered to the pollinator is a site for its brood development. During oviposition, female pollinating wasps deposit their eggs into the ovaries of some flowers, inducing the formation of galls where their offspring subsequently develop [15]. In addition to the pollinating wasps, several non-pollinating fig wasp species (NPFW), belonging to several Chalcidoidea families (e.g., Epichrysomallidae, Eurytomidae, Pteromalidae and Torymidae), also utilize figs to rear their broods [16]. These NPFWs belong to diverse ecological guilds, including gall-inducers, kleptoparasites (i.e., phytophagous species unable to induce their galls, so they occupy galls induced by other wasps), and parasitoids, which oviposit inside other wasps’ galls and consume the host larvae [17,18,19]. Thus, the successful functioning of the fig–wasp mutualism relies not only on floral traits typically associated with pollinator attraction but also on additional specialized adaptations facilitating the larval development of the associated wasps.

In various fig tree species, irregularly shaped regions known as maculae are visible on the external surface of the fig. Depending on the species, maculae may exhibit either a lighter or darker coloration compared to the typical pigmentation of the fig’s surface [20,21,22,23]. Notably, these structures concentrate most of the stomata present on the fig’s surface [24]. The precise function of fig maculae is not yet fully understood; however, field observations on two Southeast Asian fig species belonging to section *Sycocarpus* (*Ficus schwarzii* Koord. and *F. benguetensis* Merr.) demonstrated that this structure functions as extrafloral nectaries (EFN), mediating interactions with ants that patrol and protect figs against herbivores, even though the authors did not explicitly refer to it as a macula [25,26]. Despite these ecological roles, structural evidence of nectar-secreting cells in fig maculae has not been investigated in these or other *Ficus* species to date.

Given the specific characteristics of brood-site pollination, figs function as nurseries that shelter hundreds to thousands of developing wasp larvae. Under these conditions, maintaining a stable internal microenvironment becomes critical, as fig temperature can rise not only due to external factors such as solar radiation but also because of endogenous processes associated with cellular respiration within the fig [13]. Patiño et al. [27] experimentally demonstrated that internal fig temperatures at or above 35 °C are lethal to immature wasps. In the same study, they showed that transpiration is responsible for the fig cooling, preventing it from reaching critical temperatures when fully exposed to sunlight. Thus, a potentially relevant issue is the maintenance of internal fig temperature within limits tolerable for insect development. Gas exchange, particularly oxygen uptake required for larval respiration and elimination of carbon dioxide produced by the larvae, is probably another fundamental aspect of this interaction. In this context, we investigated the maculae’s morphological structure and figs’ ecological aspects at different phases of the fig reproductive cycle to infer the functional role of maculae in *Ficus citrifolia* Mill. (section *Americanae*). Specifically, our morphological analyses focus on the secretory function of maculae and their potential connection in gas exchange processes related to larval respiration and thermal regulation within figs.

## 2. Results

### 2.1. Morphological Aspects

**Figure 1:**
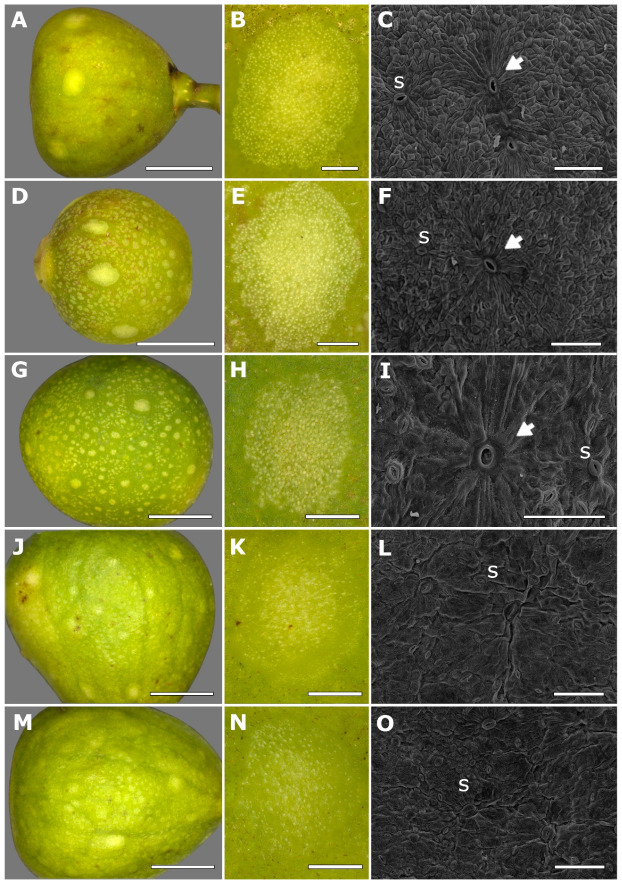
Morphological structure of fig maculae in *Ficus citrifolia* across different phases of the fig reproductive cycle (A–E rows 1 to 5, respectively), following the classification of Galil & Eisikowitch [13]. Left column (**A**,**D**,**G**,**J**,**M**): figs at phases pre-female (top) to postfloral (bottom) showing the distribution of maculae on the fig surface. Middle column (**B**,**E**,**H**,**K**,**N**): close-up views of individual maculae; stomata are visible as small punctate structures across the surface. Right column (**C**,**F**,**I**,**L**,**O**): scanning electron micrographs of maculae surfaces showing the central pore (arrow) and surrounding stomata (s). Scale bars: (**A**,**D**,**G**,**J**,**M**) = 5 mm; (**B**,**E**,**H**,**K**,**N**) = 500 µm; (**C**,**F**,**I**,**L**,**O**) = 100 µm.

In *F. citrifolia*, fig maculae are slightly protuberant structures with a lighter coloration than the surrounding fig surface regions. Such a light appearance is due to the high density of stomata on the maculae, which are visible under higher magnification (Figure 1). Maculae are distributed across the entire surface of the fig, with small ones (approximately 0.2 to 0.5 mm in diameter) occurring in large numbers. Less numerous, larger maculae (approximately 1.5 to 2 mm in diameter) are concentrated on the distal half of the fig (Figure 1A,D). Both the maculae and the non-macula regions lighten in color during the final phases of the fig reproductive cycle (Figure 1J,M). Histochemical tests using neutral red revealed high metabolic activity in maculae cells during phase B (Figure S1).

The macula has a central large pore surrounded by stomata, which are randomly distributed over the macula surface at an approximate density of 300 stomata/mm^2^, contrasting with a density of approximately 13 stomata/mm^2^ outside the maculae (Figure S2). The central pore differs from a stomatum by its larger opening and by lacking guard cells. In fig phases A to C, stomata are more conspicuous compared to those on the surrounding fig epidermis (Figure 1C,F,I,L,O).

**Figure 2:**
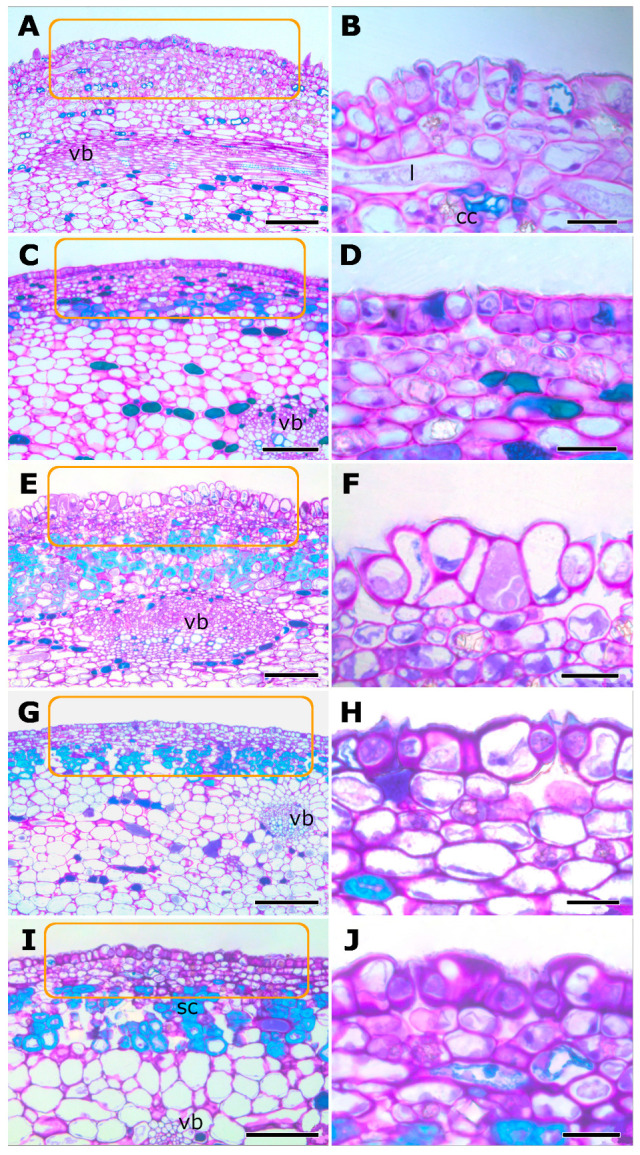
Histological structure of the fig macula in *Ficus citrifolia* throughout different phases (phases **A**–**E**, *sensu* Galil and Eisikowitch [13]) of the fig reproductive cycle (**A**,**C**,**E**,**G**,**I**). Longitudinal sections showing the macular region (rectangles). Right-hand panels (**B**,**D**,**F**,**H**,**J**) indicate magnified areas in the left-hand panels. Each pair of images in a row represents a phase of the fig reproductive cycle. Note that maculae show a similar anatomical organization across all phases, with variations related to epidermal cell rounding, subepidermal wall thickening, and increased secretory content. It consists of a single layer of cuticularized, rounded epidermal cells, papillose from phase B onward, with several stomata. Subepidermal layers contain small, densely packed parenchymatic cells, laticifers (l), and crystalliferous cells (cc). From phase C (**C**,**D**), some subepidermal cells differentiate into lignified sclereids (sc). Phenolic cell number increases from phases (**A**–**D**), peaks in C (**E**,**F**), and decreases in later phases (**G**–**J**). Note the vascular bundles (vb) near the macula in all images on the left. Scale bars: (**A**,**C**,**E**,**I**) = 100 µm; G = 200 µm; (**B**,**D**,**F**,**H**,**J**) = 20 µm.

Histological sections indicate that the macula is composed of several cell layers. The epidermis and subepidermal regions of the macula do not differ substantially from adjacent epidermal areas. Epidermal cells are cuticularized and arranged in a single layer, whereas the subepidermal layers consist of small, densely packed parenchymatic cells, distinct from the larger epidermal cells (Figure 2). Some of these parenchymatic cells differentiate into lignified sclereids during phase C (Figure 2). Additionally, laticifers, crystalliferous and phenolic cells occur within the subepidermal region. The number of phenolic cells progressively increases in the early phases of the fig reproductive cycle, reaching its peak at phase C and subsequently decreasing in later phases (Figure 2). The macula is vascularized by ramifications of vascular bundles from the fig itself (Figure 2).

**Figure 3:**
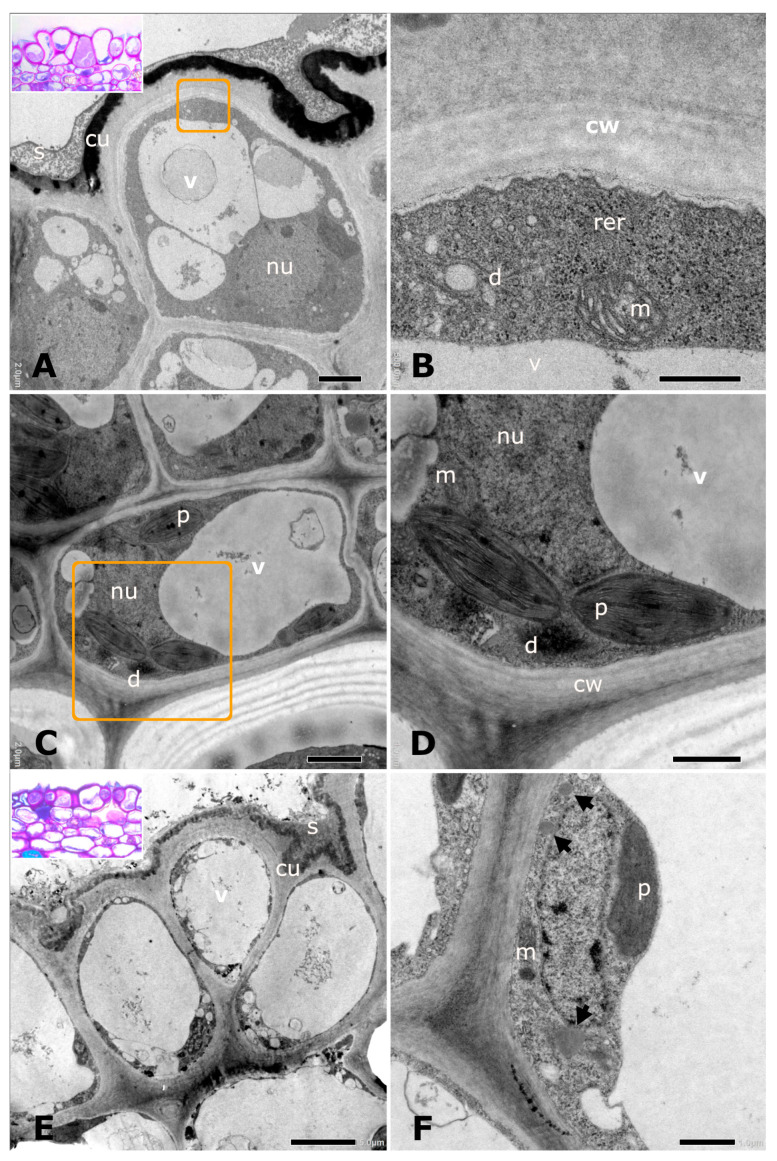
Ultrastructure of macula epidermal cells in *Ficus citrifolia* during the secretory (**A**–**D**) and post-secretory (**E**,**F**) phases. (**A**) Secretory cell showing a large nucleus (nu), vacuole (v), dense cytoplasm, and granular osmiophilic secretion on the subcuticle space (cu); note the particulate-osmiophilic secretion (s) on the cuticle. (**B**) Detail of the area marked in A showing a region with rough endoplasmic reticulum (rer), dictyosome (d), mitochondria (m), and the thickened cell wall (cw). (**C**) Secretory cells with a prominent nucleus (nu), vacuole (v), dictyosome (d), plastid (p), and mitochondria (m). (**D**) Enlargement of the region in C showing plastids (p) with osmiophilic droplets, dictyosome (d), nucleus (nu), mitochondria (m), and thickened cell wall (cw). (**E**) Post-secretory cell with vacuolated cytoplasm. Note that secretion is no longer visible on the cuticle. (**F**) Detail of the cell showing oleosomes (arrows), plastid (p), and mitochondria (m). Insets in (**A**,**E**) show toluidine blue-stained sections for anatomical reference. Scale bars: (**A**,**C**,**E**) = 2 µm; (**B**,**D**,**F**) = 0.5 µm.

The epidermal cells of the fig maculae exhibited ultrastructural characteristics typical of secretory activity related to the production of oils, sugars, and phenolic compounds (Figure 3A–D). Cells in the active secretory phase showed a prominent nucleus, large vacuoles, dense cytoplasm, abundant rough endoplasmic reticulum, numerous mitochondria, and dictyosomes with enlarged cisternae and numerous associated vesicles (Figure 3A–D). Plastids containing symmetrical thylakoids and osmiophilic droplets were also abundant in these secretory cells (Figure 3C,D). During the secretory phase, a granular, particulate, and osmiophilic secretion was observed on the cuticle (Figure 3A). Thickened walls were evident in these cells (Figure 3B,D). In contrast, epidermal cells in the post-secretory phase exhibited reduced cytoplasmic density, large vacuoles, and lacked visible secretion beneath the cuticle (Figure 3E). Plastids and mitochondria persisted but were fewer and less prominent compared to secretory-phase cells (Figure 3F).

**Figure 4:**
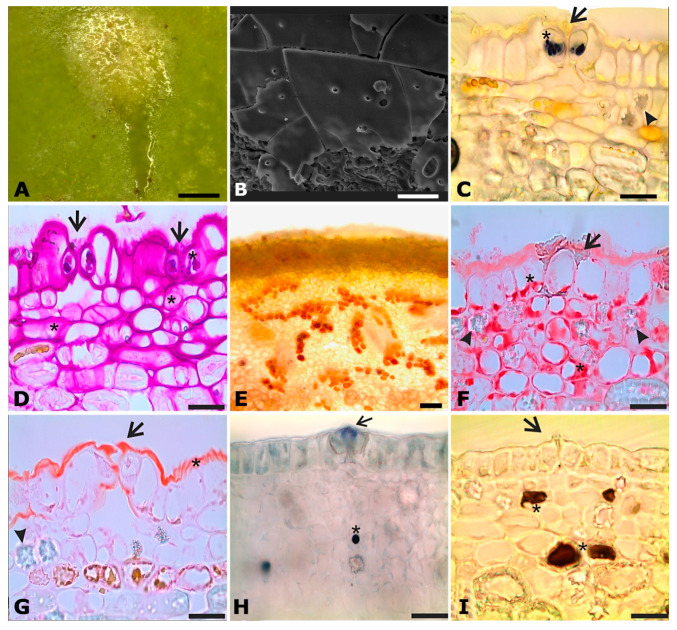
Macula secretion on a fig at the initial phase C and in situ histochemical tests on the macula of *Ficus citrifolia*. (**A**) In natura secretion; (**B**) dehydrated section on SEM; (**C**) Lugol’s reagent for starch; (**D**) PAS reagent for neutral polysaccharides; (**E**) Fehling’s reagent for reducing sugars; (**F**) Xylidine Ponceau reagent for proteins; (**G**) Sudan IV reagent for lipids; (**H**) Nadi reagent for terpenes; (**I**) Ferric chloride reagent for flavonoids. Asterisk: positive staining reaction. Arrows: stomata. Arrowheads: calcium oxalate crystals. Scale bars: (**A**) = 500 µm; (**B**) = 50 µm; (**C**,**D**,**F**–**I**) = 20 µm; (**E**) = 100 µm.

A brittle secretion with a sugary appearance when dry was observed on the maculae surface at phases B to initial C (Figure 4A,B). The secretion was more conspicuous in the early morning or on days with higher air humidity, suggesting that it evaporates rapidly under dry conditions. Histochemical tests detected substantial amounts of starch in stomatal guard cells (Figure 4C), as well as reducing sugars (Figure 4D,E), proteins (Figure 4F), and terpenic droplets (Figure 4G,H) within epidermal and subepidermal parenchymatic cells. Phenolic compounds/flavonoids were identified in epidermal cells and subepidermal cells (Figure 4I). The secretion is released to the exterior of the plant, possibly through stomata (Figure 1 and Figure 4C–H), since the cuticle (Figure 2F and Figure 4A) constitutes a barrier that prevents the diffusion of substances across the cell wall, making stomata the likely sites through which the secretion can pass.

### 2.2. Ecological Aspects

**Figure 5:**
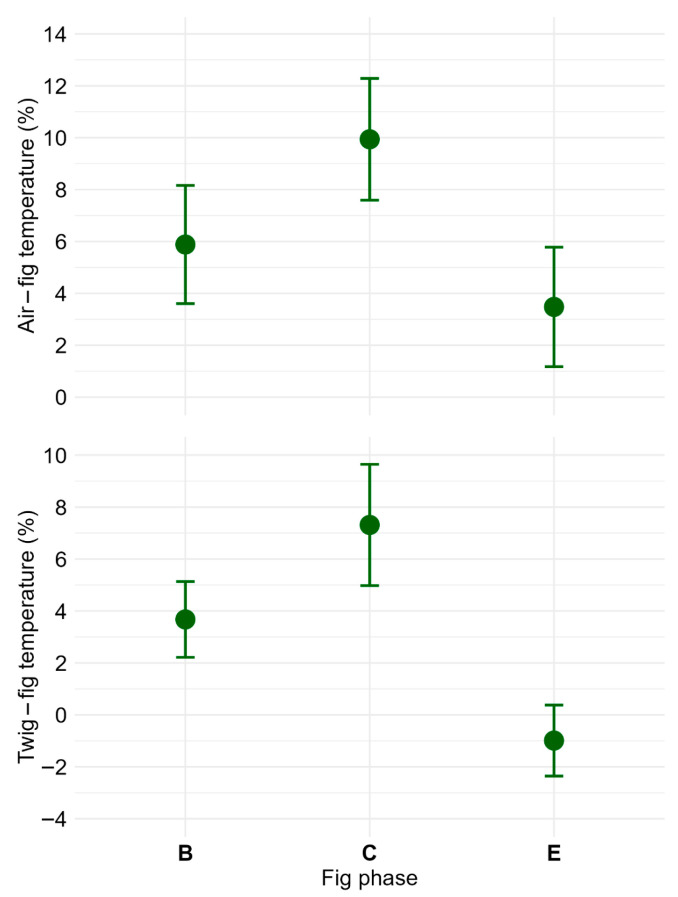
Relative temperature differences (%) between *Ficus citrifolia* figs and their environment across developmental phases B, C and E. The upper panel shows the difference between internal fig temperature and ambient air temperature (Air − Fig), while the lower panel shows the difference between internal fig temperature and the temperature of the supporting twig (Twig − Fig). Data points represent the mean percentage difference, and error bars indicate standard deviation. Sample sizes were *n* = 5, 6, and 5 figs for phases B, C and E, respectively.

Figs at stages associated with pollination (phase B) and wasp larval development (phase C) exhibited lower internal temperatures compared to those in phase E, when wasps had completed their life cycle. During phases B and C, figs were on average 6 and 10% (1.8 and 3.1 °C in absolute values) cooler than the ambient air temperature, respectively. At phase E, fig temperatures were roughly 3.5% (1.1 °C) cooler than the air temperature. Figs in phases B and C were approximately 4 and 7% (1.1 and 2.2 °C) cooler than the supporting twig, respectively. Conversely, at phase E, the temperatures of figs were on average 1% warmer than the twigs (Figure 5; Table S1). These relative and absolute temperature differences across fig phases were statistically significant (Table S2).

The internal temperature of figs coated with silicone grease was on average 1.8 ± 0.5 °C higher than that of untreated figs (mean ± SD, *n* = 12). Relative to ambient air, grease-coated figs were 1.3 ± 0.5 °C cooler, whereas control figs were 3.1 ± 0.49 °C cooler. These differences were statistically significant (paired *t*-test = 11.3, df = 11, *p* < 10^−6^).

**Figure 6:**
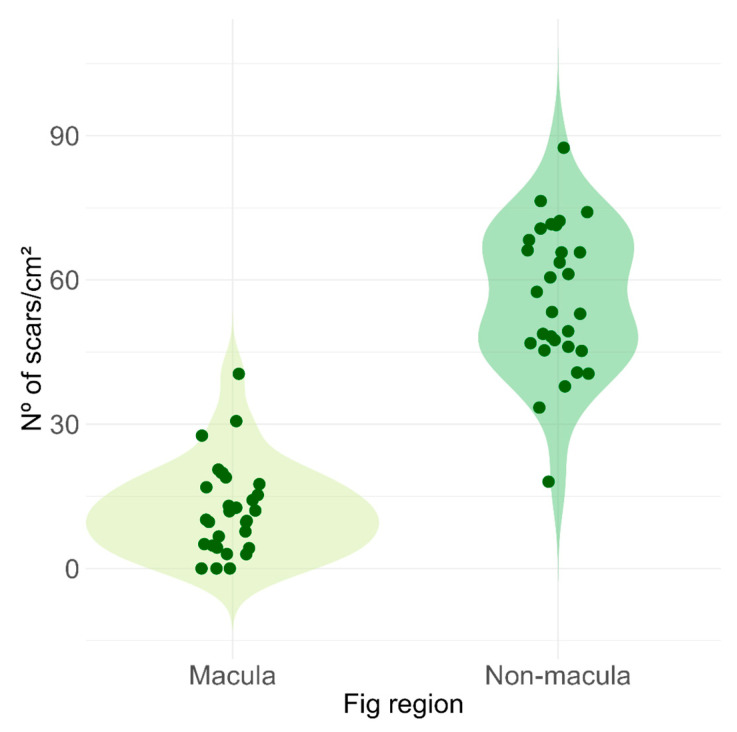
Comparison of the number of oviposition scars per cm^2^ in regions with and without epidermal maculae on the surface of *Ficus citrifolia* figs. Violin plots represent the kernel density estimation of the data distribution for each group, with individual data points overlaid. The density of scars is significantly higher in non-macula regions (paired *t*-test = 13.8, df = 29, *p* < 10^−14^).

Maculae occupied a total area of 0.25 ± 0.05 cm^2^, corresponding to approximately 5% of the fig surface, while the remaining surface without maculae measured 4.58 ± 0.52 cm^2^ (mean ± SD, *n* = 30 figs). The density of scars caused by ovipositors of non-pollinating fig wasps was approximately five times higher on fig areas without maculae compared to macula-covered areas (macula: 12.3 ± 9.5 scars/cm^2^; non-macula: 56.2 ± 15.1 scars/cm^2^; paired *t*-test = 13.8, df = 29, *p* < 10^−14^; Figure 6).

Although we did not conduct systematic observations of fig–insect interactions, we frequently observed ants patrolling the figs, particularly during phases when macular secretions were present.

## 3. Discussion

The results support our hypothesis that fig maculae play a multifunctional role in the obligate mutualism between *Ficus* species and their pollinating wasps. The secretory nature of the maculae, indicated by the presence of a sugary-like exudate (see Figure 4A,B) and the detection of reducing sugars and terpenes using histochemical tests, confirmed by the organelle population in ultrastructural analyses using TEM [28,29], and the observation of patrolling ants during the period when the maculae are actively secreting suggest that these structures may function as odoriferous nectar-secreting glands or extrafloral nectaries (EFNs) [25,26] involved in chemical signaling with insects [30]. The presence of stomata in the macula further indicates their involvement in releasing odoriferous nectar to the exterior of the plant, as occurs in most nectaries [31]. The macules of *F. citrifolia* fit the concept of non-structural nectaries because they do not exhibit a clear anatomical organization, unlike the arrangement expected in structural nectaries, which are composed of a nectariferous palisade epidermis, subepidermal nectariferous parenchyma, and vascular ramifications. Nectar in non-structural nectaries may be released through diffusion, cell rupture, or stomata [31,32,33]. These nectaries are often challenging to detect, as the secretory cells tend to be metabolically active only during specific stages of gland development. This condition is generally considered less specialized than structural nectaries, but it still plays a role in floral biology and plant–pollinator interactions.

As the macula concentrates a high density of stomata, we also propose an additional function in gas exchange, possibly associated with larval respiration and thermal regulation via fig transpiration [13,27]. Supporting this, our ecological results demonstrate that figs at phases corresponding to pollination and larval development are cooler than both ambient air and the supporting twig, and that non-pollinating fig wasps selectively avoid probing fig regions corresponding to maculae. Considering that the subepidermal tissues of the maculae do not differ substantially from those of adjacent fig regions, the observed oviposition avoidance behavior may reflect a selective pressure to prevent structural damage to maculae caused by ovipositor insertion, thus preserving their functional integrity.

A dual role in nectar secretion and emission of volatile organic compounds (VOCs) of *F. citrifolia* maculae is supported by histochemical and ultrastructural evidence of intense secretory cell metabolism, such as the presence of starch reserves, abundant mitochondria, rough endoplasmic reticulum, numerous dictyosomes, plastids containing osmiophilic droplets and oleosomes, all indicative of active synthesis and accumulation of sugars and terpenes [29,31,32]. The EFN function is further corroborated by (i) the detection of macula secretions, characterized by a sugary appearance upon dehydration and visible under SEM [34], and (ii) positive histochemical tests for polysaccharides (PAS) and reducing sugars (Fehling’s reagent) within epidermal and subepidermal cells. The hypothesis of the VOC-emission function is supported by the detection of terpenes (Nadi reagent) within subepidermal cells, which are widely associated with insect attraction [35,36,37]. The EFN-mediated interactions involving ants that patrol and protect figs against herbivores have been demonstrated in two Southeast Asian fig tree species belonging to section *Sycocarpus* (*F. schwarzii* and *F. benguetensis*) [25,26], with the presence of sugars in the secretion being chemically tested, and identified, only in figs of *F. benguetensis*. However, the relationship between nectar secretion and VOC emission remains poorly understood. Some studies suggest that VOCs emitted directly by nectar modulate the behavior of various visiting insects [38]. For example, benzyl acetone, a VOC present in floral nectar of *Nicotiana attenuata* Torr. ex S.Watson, enhances pollinator visitation rates [39]. Nectarivorous and pollinivorous mites, known as hummingbird flower mites (Gamasida: Ascidae), use nectar-derived VOCs as olfactory cues to locate their host plants [40]. In the context of EFNs, behavioral assays have shown that the parasitoid wasp, *Microplitis croceipes* (Cresson) (Hymenoptera: Braconidae), can rapidly detect EFNs on cotton plants based solely on scent, as quickly as they detect honey and significantly faster than odorless sugar solutions, suggesting that EFNs also release VOCs with an attractive function [41]. Therefore, we hypothesize that the macula secretions in *F. citrifolia* act as fragrant nectar, potentially providing nutritional rewards and chemical signaling cues for ants.

The association between fig trees and ants, which often affects the fig–fig wasp mutualism through predation on both pollinating and NPFW, is widely recognized [42]. Most reports of ant–fig associations involve an indirect interaction mediated by homopterans, where ants are attracted by the sugary secretions (honeydew) produced by these insects and, by protecting homopterans against their natural enemies, parallelly protect figs from externally ovipositing NPFWs [42,43]. Direct evidence for ant predation on NPFWs includes field observations of amputated ovipositors embedded in fig surfaces [44] and experimental manipulations demonstrating that excluding ants from fig access increases oviposition rates by NPFWs, consequently increasing parasitism rates of pollinating wasps and seeds [43,45]. In contrast, direct associations involving ants attracted to fig maculae that function as EFNs are, to the best of our knowledge, restricted to *Ficus* species belonging to the section *Sycocarpus* [25,26]. Therefore, our results suggest that the EFN role of maculae might be more widespread within the *Ficus* than previously recognized. An interesting open question for future studies is whether the association with ants mediated by conspicuous EFNs, as observed in *F. schwarzii* and *F. benguetensis*, and the association mediated by homopterans represent alternative and mutually exclusive evolutionary strategies.

In the fig–fig wasp mutualism, two physiological aspects are likely critical for ensuring the viability of the interaction: gas exchange (i.e., oxygen uptake required for larval respiration and elimination of carbon dioxide produced by the larvae), and the maintenance of internal fig temperature within limits tolerable for insect development. Internal fig temperatures may rise due to external factors such as solar radiation, and potentially due to endogenous heat generated by cellular respiration within figs. Although empirical data on endogenous heating in figs are not currently available, this hypothesis is plausible given that thermogenic plants are known to generate considerable heat via elevated respiratory rates [12,13]. Our results demonstrated that blocking fig transpiration (using silicone grease) resulted in an average increase of 1.8 °C compared with figs that were able to transpire. Further support for the hypothesis that maculae function in fig thermal regulation comes from experimental evidence demonstrating the detrimental effects of increased fig temperature on pollinator wasp survival [27]. In that study, the authors showed that when transpiration was artificially blocked, internal fig temperatures rose substantially, reaching lethal values for wasp larvae within two hours. These results suggest that thermal regulation is likely to be especially relevant in fig species with larger figs. Our results showed that *F. citrifolia* figs at the phase corresponding to wasp larval development (phase C) were, on average, 7% cooler than the twigs supporting them. In contrast, after the emergence of the wasps (phase E), fig and twig temperatures nearly converged. Thermal regulation, therefore, represents a compelling aspect for future research opportunities, including investigations of how fig size, which varies greatly among species, is potentially influenced by environmental conditions (e.g., temperature and humidity) or by biotic constraints related to pollen and seed dispersal strategies.

Thermal regulation mediated by fig transpiration allows us to speculate that the brood-site pollination mutualism between figs and fig wasps involves a considerable water demand associated with maintaining suitable thermal conditions necessary for wasp larval development. Freestanding fig species (i.e., those growing directly from the soil without an initial host tree) belonging to the section *Pharmacosycea* typically occur in moist habitats or are restricted to riparian forests [46,47,48,49], suggesting a strong dependence on water availability. In regions characterized by highly seasonal climates and prolonged dry seasons, such as southeastern Zimbabwe, several fig species are similarly restricted to riparian zones [50]. This putative reliance on water availability could be associated with evolutionary adaptations that enhance water acquisition capacities in some canopy-emergent hemiepiphytes species in section *Americanae*. For instance, trees of *F. schultesii* Dugand and *F. eximia* Schott develop extensive superficial roots, reaching lengths of up to 100 m, enabling large-scale water foraging [49,51]. At the landscape scale, evidence from semi-deciduous seasonal forests indicates that water availability, measured as stream density, is the most critical determinant of both fig species richness and density [49]. Therefore, our study suggests that fig maculae represent a key adaptation in fig trees, with implications extending beyond individual plants to influence fig population dynamics and community assembly.

## 4. Materials and Methods

### 4.1. Study Species and Site

The study was conducted between January 2023 and May 2025 on the University of São Paulo campus in Ribeirão Preto, Brazil (21.166260° S, 47.855183° W). The campus landscape consists of gardens and lawns with a mix of spontaneous and planted tree species. *Ficus citrifolia*-*guaranitica* form [52] is a medium-sized, pioneer, monoecious species that are commonly established as a hemi-epiphyte on trees or artificial structures, often thriving in disturbed environments. Its figs develop in pairs at the leaf axils and reach 1.5–2.5 cm in diameter when fully ripe.

### 4.2. Morphological Aspects

We sampled figs of *F. citrifolia* at all five stages of the reproductive cycle (phases A–E), as defined by Galil and Eisikovitch [15]. Phase A represents initial fig growth, corresponding to pre-anthesis of pistillate flowers. Phase B is characterized by anthesis of pistillate flowers, during which figs release volatile compounds that attract pollinating wasps [36,53]. Phase C corresponds to the development of larvae and seeds, as well as the period during which most non-pollinating fig wasps (NPFWs) oviposit within figs. Phase D is marked by the anthesis of staminate flowers and coincides with the maturation of wasps into adults. At this stage, female pollinator wasps load pollen and exit the fig, subsequently searching for receptive figs (phase B) on conspecific trees. Phase E corresponds to fig ripening, during which figs become attractive to frugivores.

Voucher specimens are deposited at the SPFR herbarium under registration numbers 0014969 and 012997.

For surface and anatomical analyses of fig maculae, figs were fixed in neutral buffered formalin for 24 h [54], washed in phosphate buffer, and gradually dehydrated through an ethanol series.

Surface morphology of fig maculae was observed under a Leica MZ16 stereomicroscope (Leica Microsystems, Wetzlar, Germany) and a Jeol JSM-6610LV (Jeol, Tokyo, Japan) scanning electron microscope (SEM). For SEM analyses, fixed fig samples were dehydrated through a graded ethanol series, dried using a CO_2_ critical-point dryer (Bal-Tec CPD 030) (BalTec, Pfäffikon, Switzerland), mounted on metal stubs with double-sided carbon adhesive tape, and coated with gold for 300 s in a Bal-Tec SCD 050 sputter-coater (BalTec, Pfäffikon, Switzerland).

Anatomical characteristics of maculae were investigated using fixed samples dehydrated in a graded ethanol series, embedded in Leica historesin [55], sectioned longitudinally at 3.5 µm thickness using a rotary microtome, and stained with 0.05% toluidine blue at pH 6.8 [56]. Observations were carried out under a light microscope. Histochemical localization was performed on either fixed samples or fresh material collected between 08:00 and 09:00 a.m. Sections were subjected to histochemical reactions using Lugol’s reagent for starch detection [57], periodic acid–Schiff (PAS) reagent for polysaccharides [58], Fehling’s reagent for reducing sugars [59], Ponceau xylidine for proteins [60], Sudan IV for lipids [61], Nadi reagent for terpenes and oleoresins [62], and ferric chloride for phenolic compounds [59]. Photodocumentation was performed using a Leica DM 4500 B microscope equipped with a Leica DFC 320 digital camera (Leica Microsystems, Wetzlar, Germany).

Ultrastructural characteristics of macula cells were analyzed after the samples were fixed in Karnovsky’s solution (0.075 M in phosphate buffer, pH 7.2–7.4, for 4 h) [63], post-fixed with osmium tetroxide (1% in the same buffer for 1 h), dehydrated in an acetone series, and embedded in Araldite. Ultrathin sections were stained with 2% uranyl acetate for 15 min [64], followed by lead citrate for 15 min [65], and then examined and documented using a Philips EM 208 electron microscope (Koninklijke Philips, Amsterdam, The Netherlands).

### 4.3. Ecological Aspects

To investigate the putative role of maculae in fig thermal regulation, internal temperatures of the figs and their supporting twigs were measured during the hottest period of the day (12:00–14:00 h). Simultaneously, air temperature in the shade was recorded as a control. Measurements were performed on figs at phases B, C and E. Two metallic temperature sensors, each 4 mm in diameter, were used to simultaneously record internal fig/twig and ambient air temperatures. To minimize interference from ambient temperature on internal measurements, the portion of the metallic sensor that remained exposed outside the fig or twig was covered with a piece of styrofoam (Figure S3). Data were collected every 30 s over a period of 20 min using a LogBox-AA Novus data logger (Novus, Canoas, Brazil). Due to the initial temperature stabilization period following sensor insertion, the first 10 min of data collection were excluded from analysis. Temperatures were measured in 5, 6, and 5 figs (and their respective twigs) at developmental phases B, C, and E, respectively.

To evaluate the effect of fig transpiration on thermal regulation, we conducted an experiment in which the internal temperatures of phase C figs coated with silicone grease, to block transpiration, and untreated figs (control) were measured simultaneously, along with ambient air temperature. Measurements were performed in the same manner as described for the previous experiment. Following the application of silicone grease, a 30 min interval was allowed before starting the temperature recordings to ensure stabilization of the fig temperature. Temperatures were then recorded every 30 s over a period of 20 min, with the first 10 min excluded from analysis. Measurements were taken on 12 pairs of figs (treatment and control) during the hottest period of the day (12:00–14:00 h). Differences between air temperature and fig temperature for grease-coated and control figs were compared using a paired *t*-test.

**Figure 7:**
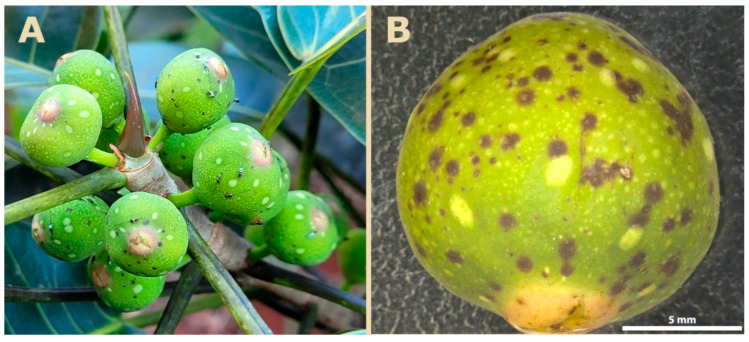
Oviposition scars on the surface of *Ficus citrifolia* figs. (**A**) Individuals of the non-pollinating fig wasp genus *Idarnes* probing the fig surface. (**B**) Numerous oviposition scars on a phase C fig. Note that most scars are concentrated in regions lacking maculae.

During the study, we observed scars on the fig surface resulting from the insertion of ovipositors by NPFWs (Figure 7). These scars appeared to be less frequent within the macula regions compared to areas without maculae. To quantitatively test this observation, we compared the density of scars in macula and non-macula areas. We hypothesized that oviposition scars could potentially interfere with the functional role of maculae, particularly their involvement in gas exchange and/or thermal regulation of the figs. Thus, a behavior by wasps of avoiding maculae during oviposition might indicate a functional role of these structures in supporting larval development.

The density of oviposition scars (scars per cm^2^) was estimated separately for macula and non-macula areas on the fig surface. The total surface area of the fig (FA) was calculated from its diameter using the formula for the surface area of a sphere, subtracting the ostiolar area, approximated as a circular region. The area occupied by maculae (MA) was calculated by summing the individual macula areas on each fig. Because maculae range from circular to elliptical shapes, their areas were estimated using the ellipse area formula, *A* = *π* × *a* × *b*, where *a* and *b* represent the major and minor axes, respectively. Subsequently, the non-macula area (NMA) was estimated as NMA = FA − MA. Oviposition scars were counted on 30 figs collected from two individual trees (10 and 20 figs, respectively). The densities of oviposition scars within macula versus non-macula areas were statistically compared using a paired *t*-test.

## Data Availability

The data supporting the findings of this study are available from the corresponding authors upon reasonable request. The data are not publicly available because they form part of a larger ongoing research project, and public dissemination at this stage could compromise the ability to develop and publish further findings.

## Supplementary Materials

The following supporting information can be downloaded at: https://www.mdpi.com/article/10.3390/plants14182885/s1, Figure S1. Macula on a phase B fig of *Ficus citrifolia* subjected to the neutral red test. Figure S2. Method used for stomatal counting in the macula of *Ficus citrifolia* figs at developmental phase B. Figure S3. The temperature measurement setup used in the study. Table S1. Mean temperature (±standard deviation) of the air, fig, and supporting twig measured during the hottest period of the day (12:00–14:00 h) in *Ficus citrifolia* figs at developmental phases B, C, and E. Table S2. Mean relative and absolute temperature differences (±standard deviation) between figs and the ambient air (Air − Fig), and between figs and their supporting twigs (Twig − Fig) at developmental phases B, C, and E of *Ficus citrifolia*.

## Abbreviations

The following abbreviations are used in this manuscript:

NPFW	Non-Pollinating Fig Wasp
SEM	Scanning Electron Microscope
TEM	Transmission Electron Microscope
EFN	Extrafloral Nectarie
VOC	Volatile Organic Compound
SPFR	Herbário do Departamento de Biologia da Faculdade de Filosofia, Ciências e Letras de Ribeirão Preto. Universidade de São Paulo
