# Supplementary material for: Fig Macula as a Key Multifunctional Structure Mediating the Fig–Fig Wasp Mutualism
Source: Plants (Basel). 2025 Sep 17;14(18):2885. doi: 10.3390/plants14182885 (PMC12473752; doi:10.3390/plants14182885)
Supplement: Supplementary file 1 [file plants-14-02885-s001.zip › plants-3818096-supplementary/plants-3818096-supplementary.pdf]

plants

Supplementary Material

MDPI

Article

Fig macula as a key multifunctional structure mediating the fig–fig wasp mutualism

Simone Pádua Teixeira [1], Jackeline Varanda Silva [1,2], Vitor Cassius Santos [1,2], Luan Mazzeo [2], Rayssa Conceição Coelho Correa [2] and Rodrigo Augusto Santinelo Pereira [2,*]

[1] Departamento de Ciências Farmacêuticas, Faculdade de Ciências Farmacêuticas de Ribeirão Preto, Universidade de São Paulo, Ribeirão Preto, SP, 14040-903, Brazil
[2] Departamento de Biologia, Faculdade de Filosofia, Ciências e Letras de Ribeirão Preto, Universidade de São Paulo, Ribeirão Preto, SP, 14040-130, Brazil
* Correspondence: raspereira@usp.br

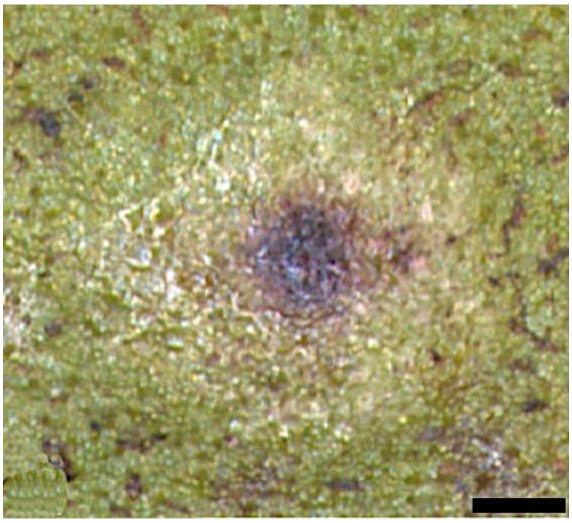

Figure S1. Macula on a phase B fig of *Ficus citrifolia* subjected to the neutral red test. The positive staining indicates intense metabolic activity in the macula cells. Scale bar = 200 μm.

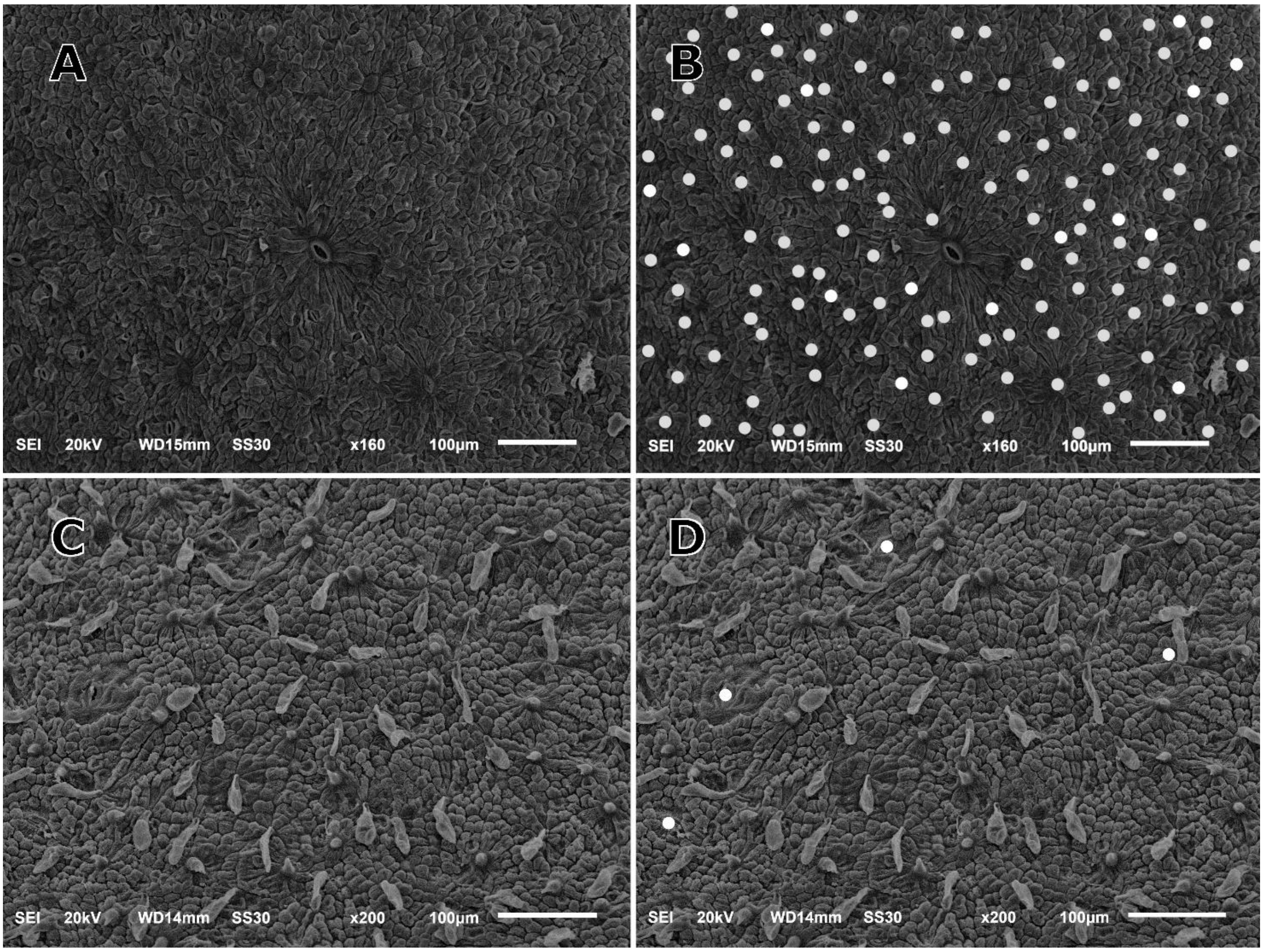

Figure S2. Method used for stomatal counting in the macula of *Ficus citrifolia* figs at developmental phase B. (A) Original scanning electron microscopy (SEM) image of the macula surface. (C) Original SEM image of the surface outside the macula. (B and D) The same pictures, with stomata marked by white circles, for quantification using ImageJ software (version 1.54g). For the macula region, a total of 145 stomata were identified within the sampled area of 0.44 mm², resulting in a stomatal density of 329.5 stomata/mm². In contrast, for the non-macula region, four stomata were observed within an area of 0.309 mm², representing 12.9 stomata/mm².

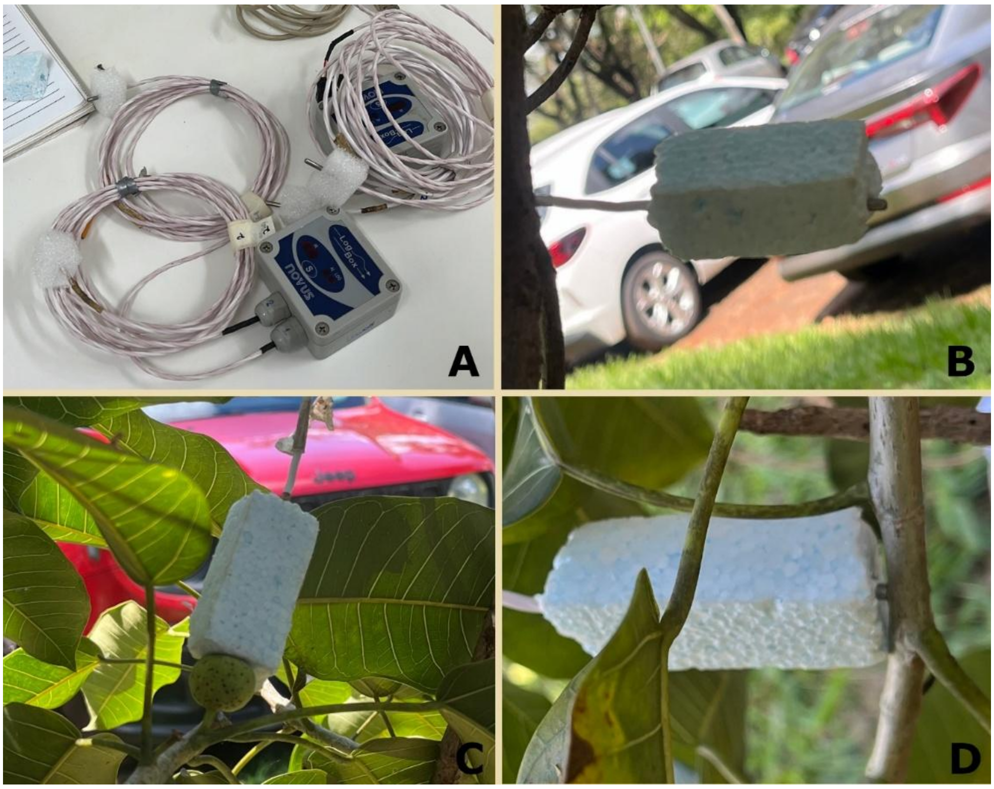

Figure S3. The temperature measurement setup used in the study. (A) LogBox-AA Novus data logger and metallic temperature sensors. (B) Air temperature measurement in the shade, where only the tip of the sensor, equivalent in length to the portion inserted into figs or twigs, was left exposed. (C) Internal temperature measurement in the fig. (D) Internal temperature measurement in the supporting twig. In all cases, the exposed portion of the sensor was covered with a piece of styrofoam to minimize the influence of ambient temperature on internal measurements.

Table S1. Mean temperature (± standard deviation) of the air, fig, and supporting twig measured during the hottest period of the day (12:00–14:00 h) in *Ficus citrifolia* figs at developmental phases B, C, and E. Differences between measurements are shown in absolute values (°C) and as percentages (%), with positive values indicating that the first element in the pair is warmer than the second (e.g., Air–Fig). n = sample size.

Phases	n	Air (°C)	Fig (°C)	Twig (°C)	Air-Fig (°C)	Air-Twig (°C)	Twig-Fig (°C)	Air-Fig (%)	Air-Twig (%)	Twig-Fig (%)
B	5	30.3 ± 3.0	28.5 ± 2.3	29.6 ± 2.8	1,8 ± 0,8	0,7 ± 0,6	1,1 ± 0,5	5.9 ± 2.3	2.3 ± 1.7	3.7 ± 1.5
C	6	31.0 ± 0.2	27.9 ± 0.6	30.1 ± 0.4	3,1 ± 0,7	0,9 ± 0,5	2,2 ± 0,7	9.9 ± 2.3	2.8 ± 1.7	7.3 ± 2.3
E	5	29.7 ± 2.5	28.6 ± 2	28.3 ± 2.1	1,1 ± 0,7	1,3 ± 0,5	-0,3 ± 0,4	3.5 ± 2.3	4.4 ± 1.3	-1.0 ± 1.4

Table S2. Mean relative and absolute temperature differences (± standard deviation) between figs and the ambient air (Air − Fig), and between figs and their supporting twigs (Twig − Fig) at developmental phases B, C, and E of *Ficus citrifolia*. The lower row shows the results of one-way ANOVA followed by Tukey's multiple comparison test (5% significance level). Different lowercase letters indicate statistically significant differences among phases. n =sample size.

Phases	n	Air - Fig (%)	Twig - Fig (%)	Air - Fig (°C)	Twig - Fig (°C)
B	5	5.9 ± 2.3[b,c]	3.7 ± 1.5[b]	1,8 ± 0,8[b,c]	1,1 ± 0,5[b]
C	6	9.9 ± 2.3[a]	7.3 ± 2.3[a]	3,1 ± 0,7[a]	2,2 ± 0,7[a]
E	5	3.5 ± 2.3[c]	-1.0 ± 1.4[c]	1,1 ± 0,7[c]	-0,3 ± 0,4[c]
ANOVA		$F_{2,13} = 11.1$, $P = 0.0016$	$F_{2,13} = 28.3$, $P < 10^{-4}$	$F_{2,13} = 10.1$, $P = 0.002$	$F_{2,13} = 26.1$, $P < 10^{-4}$
